# Pipeline Leak Detection: A Comprehensive Deep Learning Model Using CWT Image Analysis and an Optimized DBN-GA-LSSVM Framework

**DOI:** 10.3390/s24124009

**Published:** 2024-06-20

**Authors:** Muhammad Farooq Siddique, Zahoor Ahmad, Niamat Ullah, Saif Ullah, Jong-Myon Kim

**Affiliations:** 1 Department of Electrical, Electronics and Computer Engineering, University of Ulsan, Ulsan 44610, Republic of Korea; mfarooq229@mail.ulsan.ac.kr (M.F.S.); zahooruou@mail.ulsan.ac.kr (Z.A.); niamat016@mail.ulsan.ac.kr (N.U.); saifuou@mail.ulsan.ac.kr (S.U.); 2 PD Technology Cooperation, Ulsan 44610, Republic of Korea

**Keywords:** continuous wavelet transforms, deep belief network, genetic algorithm, least squares support vector machine

## Abstract

Detecting pipeline leaks is an essential factor in maintaining the integrity of fluid transport systems. This paper introduces an advanced deep learning framework that uses continuous wavelet transform (CWT) images for precise detection of such leaks. Transforming acoustic signals from pipelines under various conditions into CWT scalograms, followed by signal processing by non-local means and adaptive histogram equalization, results in new enhanced leak-induced scalograms (ELIS) that capture detailed energy fluctuations across time-frequency scales. The fundamental approach takes advantage of a deep belief network (DBN) fine-tuned with a genetic algorithm (GA) and unified with a least squares support vector machine (LSSVM) to improve feature extraction and classification accuracy. The DBN-GA framework precisely extracts informative features, while the LSSVM classifier precisely distinguishes between leaky and non-leak conditions. By concentrating solely on the advanced capabilities of ELIS processed through an optimized DBN-GA-LSSVM model, this research achieves high detection accuracy and reliability, making a significant contribution to pipeline monitoring and maintenance. This innovative approach to capturing complex signal patterns can be applied to real-time leak detection and critical infrastructure safety in several industrial applications.

## 1. Introduction

Pipelines offer the most cost-effective means of transporting critical resources such as water, gas, and oil, but long-term exposure to severe conditions can result in leaks and cracks, creating environmental pollution and incurring financial losses [1,2]. Balancing affordability with environmental responsibility is a key challenge in the management of pipeline infrastructure [3,4]. Early detection of leaks is essential to minimizing harm. Fortunately, recent technological advancements provide promising solutions. For example, machine learning algorithms are creating opportunities to improve leak-detection accuracy and efficiency [5,6]. Various monitoring strategies, such as time-domain reflectometry, vibration analysis, pressure wave methods, and acoustic emission (AE) technology [7,8,9]. Among these, AE technology is particularly promising due to its real-time sensitivity to leaks. However, cost-effectiveness remains a concern, and repair techniques such as clamps and encapsulation collars are favored over complete pipeline replacement whenever possible. Recognizing the need for both environmental protection and economic viability, this study proposes an innovative approach to leak detection by combining AE technology and machine learning algorithms [10]. This method aims to offer prompt leak detection, a baseline for accurate leak localization, and minimal false alarms while combining with existing systems and making use of reliable, low-maintenance sensors. By striking a balance between cost-effectiveness and environmental responsibility, this proposed leak detection system can significantly improve pipeline safety and lifetimes [11,12].

Extensive research on pipeline leak detection has explored numerous possible solutions, including vision systems, transient models, and sensors [13]. These endeavors collectively form the basis for pipeline health management [14,15]. Over the past decade, feature extraction and the development of artificial intelligence (AI) recognition models have been the primary focus for diagnosing pipeline health. Using advanced techniques rooted in AI and machine learning, such models analyze AE data and identify patterns indicative of leaks [16,17,18]. Elforjani et al. [19] and Banjara et al. [20] used AE technology, waveform indicators, and support vector machines (SVMs) to detect pipeline leaks. Multiscale analysis, the Kolmogorov–Smirnov (KS) test, and a Gaussian mixture model can be integrated and used to assemble a comprehensive pipeline health index [21]. An AE-based pipeline leak indicator using wave features combined with a two-sample KS test proposed by Kim et al. [22] outperformed indicators based on traditional features. A hybrid feature vector that unified AE features in time and frequency domains, as described by Xu et al. [23] improved leak detection through cross-entropy and the use of artificial neural networks (ANNs). Empirical and variational mode decompositions can also be used to pinpoint leaks [24]. Despite these advances, outstanding challenges include potential false alarms from noise in AE signals, the need for human expertise in feature extraction, and the extreme interpolation and mode mixing associated with empirical mode decomposition [25]. Previous studies demonstrated that these techniques can improve pipeline diagnoses; however, each has its limitations.

In a recently reported development, a continuous wavelet transform (CWT) was used to improve the accuracy of leak detection. A robust technique for leak detection based on AE signals has also been introduced, using a CWT to generate AE images depicting time-frequency scales [26]. The resulting scalograms are processed by a convolutional autoencoder (CAE) and ANN to extract features in local and global domains. Combining these multidomain features into a single vector enhances leak-detection accuracy. Sajjad et al. [27] also used CWT scalograms to improve overall leak detection. However, depending exclusively on CWT and AE signals to detect leaks requires a trade-off between time and frequency resolution and finding solutions to the computational challenges that come with high dimensionality. The selection of wavelet function and parameters is also important and may affect performance. To address these issues, complementary techniques and hybrid approaches can improve efficiency, robustness, and accuracy in pipeline leak detection.

Pipeline leaks can compromise material integrity, resulting in structural discontinuities, fatigue ruptures, and cracks due to stress and corrosion. These failure modes can disturb fluid flow [28]. However, intramolecular interactions within a fluid may allow a relatively regular flow even in the presence of such disruptions. AE events, which are transient waves resulting from variations in structural integrity [29]. These stress waves are detected by AE sensors placed on a pipeline. The combination of stress waves and leaks gives rise to transient “AE hits” or “AE events” [26,28]. Establishing a threshold above the background noise level is important for extracting essential AE parameters, including counts, rise, and decay time. However, relying solely on a predefined threshold to extract AE characteristics may result in false alarms due to noise [30]. Human expertise is needed to discern AE features and set an appropriate threshold for background noise to ensure accurate leak detection [31,32].

For this purpose, a new method is proposed in this study, consisting of a signal processor and deep learning algorithms to create CWT scalograms, with a Gaussian filter used to eliminate essential noise from AE signals. To further preprocess the CWT scalograms, non-local means (NLMs) and adaptive histogram equalization (AHE) not only remove the background noise of the scalograms but also enhance the information contrast of these scalograms. These new scalograms are termed enhanced leak-induced scalograms (ELIS). The proposed model’s performance was tested and verified using a dataset from a steel pipe. The primary contributions and novelty of the proposed approach can be summarized as follows:

(1) The proposed study introduces a comprehensive model that uses CWT images that are filtered using advanced NLM and AHE. The resulting scalograms are termed ELIS.
(2) A framework consisting of a deep belief network (DBN) and genetic algorithm (GA) carefully extracts informative features, while a least squares support vector machine (LSSVM) classifier accurately classifies leak and non-leak conditions.
(3) The proposed approach enhances leak discrimination by successfully differentiating among patterns related to leaks and background noise. This improves accuracy and reliability in identifying leaks, particularly in a noisy environment.
(4) To demonstrate the feasibility and effectiveness of the method in the real world, the proposed technique was tested on a steel pipe. The accuracy of the results highlights their potential for real-world applications.

In Section 2, we present the suggested technique, and Section 3 supplies the technical context essential for the investigation. Section 4 elaborates on the data-collection process and experimental design for pipeline leak detection. The outcomes of the proposed system are described in Section 5, and Section 6 concludes the study and outlines potential avenues for future research.

## 2. Proposed Method

**Figure 1:**
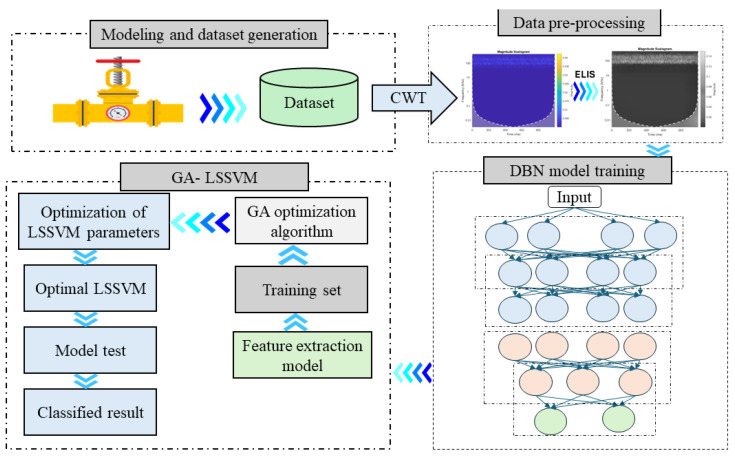
Integration of acoustic emission, image processing, and deep learning for leak detection.

Figure 1 is a flow diagram of the proposed technique, comprising the subsequent steps:

**Step 1:** A pipeline leak detection method analyzes AE signals intercepted by sensors placed strategically along the pipeline. When gas or fluid escapes from the pipeline, it generates a pressure wave that propagates through the system, leading to vibrations in the pipeline material and emissions of AE signals.

**Step 2:** A CWT is used to convert the AE signals in a time-domain into two-dimensional scalograms. These AE images represent fluctuations in energy levels across several time–frequency scales, using distinct color intensities to illustrate the changes. The scalograms effectively capture shifts in energy, employing a spectrum of colors to denote different energy levels. In the present study, the AE images serve as representations of the transformed AE signal.

**Step 3:** Filters are added to the AE images to improve the accuracy of the final output. NLM and AHE filters are applied to remove noise and increase the contrast in the scalograms, resulting in a new kind of scalogram called ELIS. As a result, accurate identification of edges is possible, leading to more accurate conclusions.

**Step 4:** The core methodology involves a DBN optimized by a GA and combined with an LSSVM for enhanced feature extraction and classification accuracy.

**Step 5:** The DBN-GA framework extracts informative features, while the LSSVM classifier identifies leak and non-leak conditions.

**Step 6**: To evaluate the performance of the proposed model, it was compared with two cutting-edge models using four main metrics: precision, accuracy, recall, and F-1 score [33].

### 2.1. Processing of Acoustic Emission Images Using a CWT

A CWT is a signal-processing method used to find the correlation between two signals using AE signals in a time-series domain [34]. A CWT employs mathematical functions distinguished by their localized properties in the time and frequency domains [35]. Using adjustable windows to transform the signals, CWT offers varying resolutions at different time periods and frequencies. This allows CWT to effectively capture local characteristics in the time–frequency domain within the signals [36]. Such adaptability makes CWT an appropriate choice for capturing non-stationary signals.

A CWT breaks down an obtained signal into wavelet functions, symbolized as $\varphi_{i,j}\left(t\right)$, through manipulations of the wavelet basis function *φ*(*t*) involving translations and scaling. The scale parameter i determines the position of the wavelet time–frequency window within the frequency domain. Conversely, the translation parameter j controls the time-domain wavelet position for the time–frequency window. A CWT is mathematically expressed in Equation (1).

(1) $$\varphi_{i,j}\left(t\right)=\frac{1}{\sqrt{\left|i\right|}}\varphi (\frac{t-i}{j})$$

By applying a CWT, the signal in the time domain transforms into a scale-domain representation. The relationship between scale and frequency is shown in Equation (2).

(2) $$f_{s}=\frac{f_{c}\times f_{sr}}{i}$$

where $f_{s}$ is the signal frequency, $f_{sr}$ denotes its sampling rate, and $f_{c}$ is used for the central frequency of the wavelet. Equation (2) facilitates the selection of the frequency distribution related to the signal’s scale and generates a wavelet time–frequency graph. This graph clearly portrays the spectral and temporal properties, along with the frequency–time–amplitude relationship. In the proposed approach, feature extraction relies on this wavelet time–frequency representation of the signal, enabling exploration of leak detection.

### 2.2. Non-Local Means and Adaptive Histogram Equalization

To enhance the quality of Laplacian-filtered CWT scalograms, minimize undesired noise, and improve brightness, the proposed method applies the NLM technique. This approach effectively reduces noise and enhances clarity, facilitating the identification of leaks, segmentation, and classification. NLM leverages the inherent self-similarity of mammographic images in the spatial domain to effectively remove noise [37]. By comparing gray levels at a certain point and the geometric patterns in neighboring pixels, NLM calculates a weighted average for each pixel, which optimizes discrete and noisy mammogram images. This process enhances various attributes of the image, increasing their suitability for subsequent analysis.

(3) $$NL\left[m\right]\left(x\right)=\sum_{y\in I}w\left(x,y\right).m(y)\;$$

Equation (3) assesses the similarity between pixels $\left(x\right)$ and $\left(y\right)$ within the image to determine the pixel weight $w\left(x,y\right)$. This computation adheres to the standard constraints of $0\leq w\left(x,y\right)\leq 1$, ensuring that the sum of the weights for a given pixel 'a' $(\sum b\;w\left(x,y\right))$ is equal to 1. These weights, which can be used to characterize individual pixels in the image, are derived from the extent of similarity between neighboring pixels $\left(x\right)$ and $\left(y\right)$.

(4) $$w\left(z\right)=\frac{1}{R_{sim}}\sum_{x=q}^{R_{sim}}\;(\frac{1}{{\left(2x+1\right)}^{2}})$$

**Figure 2:**
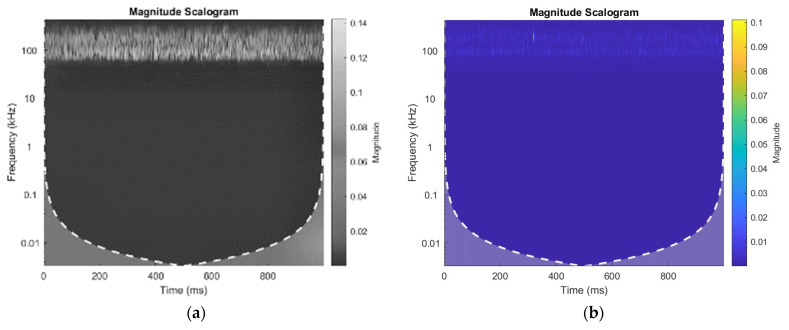
CWT Scalograms (**a**) With NLM and AHE (**b**) Without NLM and AHE.

Equation (4) calculates filter weights for neighboring pixels according to their distance from the filter center. Each pixel’s weight is influenced by its similarity (“sim”). NLM operates on the premise that image noise resembles white noise, improving images with significant redundancy. This technique smooths scalograms and enhances contrast using limited AHE [38], which targets specific sections of an image to improve contrast. Further refinement can be achieved through bilinear interpolation, ultimately enhancing the visibility and processability of mammography. Figure 2 illustrates the impact of NLM and AHE functions on CWT images, highlighting the improvements in visualization and quality. Combining these methods enhances noise reduction, contrast enhancement, and overall image refinement, indicating that ELIS is suitable for analysis and diagnosis.

### 2.3. Feature Extraction Using a Deep Belief Network Model

A DBN is a probabilistic generative model with hidden layer units and integrated restricted Boltzmann machines (RBMs) [39]. These RBMs form the building blocks of the DBN, giving it the ability to capture complex patterns in data. By stacking several layers of RBMs, DBNs can learn hierarchical representations of the input data, enabling effective feature extraction. This hierarchical structure allows DBNs to model intricate relationships within the data, making them suitable for classification and generation tasks. Overall, DBNs offer a powerful framework for unsupervised learning and feature learning [40].

**Figure 3:**
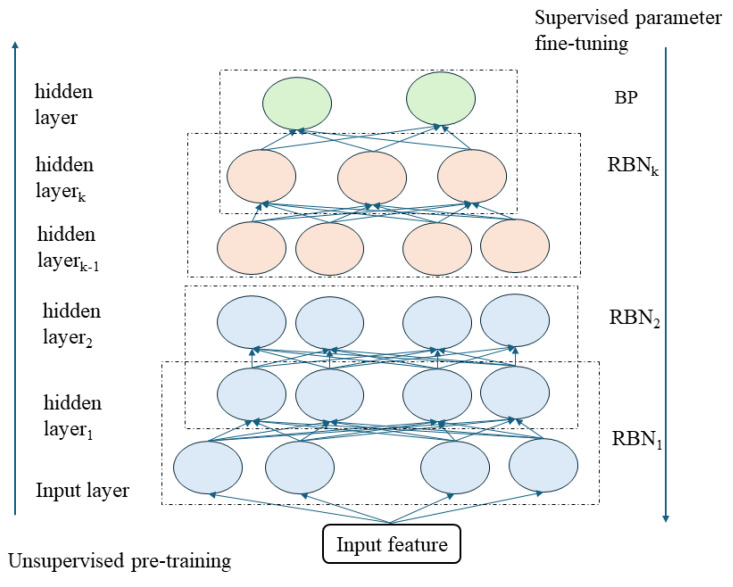
Architecture and Training Procedure of a Deep Belief Network.

Figure 3 illustrates the architecture of a DBN, with the RBM consisting of exposed e layers and hidden h layers interconnected bidirectionally. Each layer’s neurons operate independently, with the exposed input layer denoted as $e=\{e_{1},e_{2}\ldots \ldots e_{m}\}$ and the hidden layer as $h=\{h_{1},h_{2},\ldots \ldots h_{n}\}$. The RBM facilitates the learning of intricate hierarchical representations of data through unsupervised training. These representations are then used in subsequent layers of the DBN for tasks such as classification or generation. DBNs have achieved remarkable performance in various machine learning applications due to their ability to capture complex patterns in data. The probability density function between the exposed and hidden layers can be simplified as

(5) $$F\left(e,h\right)=-\sum_{a=1}^{m}e_{a}i_{a}-\sum_{b=1}^{n}h_{b}j_{b}-\sum_{a=1}^{m}\sum_{b=1}^{n}be_{a}h_{a}w_{ab}$$

where $w_{ab}$ is the connection weight between the unit a visible layer and the unit b hidden layer, while $i_{a}$ and $j_{b}$ represent the offsets of the exposed and hidden layer units, respectively.

The DBN methodology is used to extract fault features, incorporating two primary stages: pre-training and fine-tuning. During the first stage, several forms of pipeline data pass through unsupervised learning within the first layer’s RBM of the DBN. The output from this trained layer is fed into the next layers, initiating a process of layer-by-layer greedy learning that proceeds to the final layer, which yields the characteristics of the pipeline leaks. In the fine-tuning stage, supervised learning is integrated, comparing the obtained results with labeled data. Error backpropagation is used to iteratively adjust the DBN parameters and improve its performance.

One of the benefits of using a DBN is the ability to resolve complex features by training them layer by layer. This feature-extraction ability is significantly augmented in the fine-tuning phase through supervised learning, refining the model parameters for optimal performance. This method enables the extraction of crucial feature information from ELIS, providing essential support for leak diagnosis and prediction.

After the initial preprocessing of CWT images, a DBN model is deployed, as shown in Table 1. This model is designed to extract meaningful features from the preprocessed data for the binary classification tasks of identifying leak and non-leak conditions in pipelines. Constructed using TensorFlow’s Sequential API, the model comprises multiple layers, including dense layers with rectified linear unit activation, batch normalization layers, and dropout layers. These components are organized strategically to learn hierarchical data representations, with each layer capturing increasingly abstract features. Regularization is applied through L2 in dense and dropout layers to mitigate overfitting, increasing model generalizability to new data. The DBN-like architecture is trained using scaled, flattened vectors derived from the ELIS and optimized for binary classification accuracy with a binary cross-entropy loss function. After training is complete, a feature-extractor model can be obtained by replicating the DBN model up to the last dropout layer while excluding the final classification layer. This extractor is then used to make predictions, efficiently condensing higher-dimension ELIS data into meaningful feature spaces in lower dimensions. The extracted features are then used for classification using LSSVM with GA for optimization of hyperparameters. This demonstrates a comprehensive approach to pipeline leak detection, using deep learning for feature extraction and advanced machine learning algorithms for classification. The following table provides a concise summary of the DBN architecture, including the shape of each layer’s output, the activation function used, the regularization method, and the dropout rate.

The table provides a concise summary of the DBN architecture, including the shape of each layer’s output, the activation function used, the regularization method, and the dropout rate.

### 2.4. t-Distributed Stochastic Neighbor Embedding

The t-distributed stochastic neighbor embedding method is used in data analysis and visualization of complex, nonlinear relationships and patterns within datasets. This technique reduces data dimensionality, enabling the depiction of intricate structures in a more comprehensible and useful way. As a result, t-SNE has proven instrumental in revealing insights, distinguishing patterns or clusters, and interpreting fundamental connections within data. It is an efficient approach to investigating and visualizing complicated datasets or nonlinear configurations, making it an invaluable resource for data analysis and interpretation [41].

In this work, t-SNE plays an important role in improving the accuracy of the classification of the pipeline leak detection system. Feature extraction was performed on the filtered scalograms derived from the CWT, capturing valuable information about normal operations and leak-pattern states. To enhance classification accuracy and elucidate the structure of feature space, t-SNE was used to reduce the dimensionality of the feature vectors. This transformation facilitated visualization and analysis of feature cluster distribution and separation, simplifying the identification of distinct patterns related to leak and non-leak conditions. By reducing dimensionality while maintaining the interrelationships among data points, t-SNE can strengthen the classification algorithm’s capacity to distinguish patterns induced by leaks from those originating from background noise or a healthy pipeline. The resulting lower-dimensional space provides a distinct perspective on the feature clusters, enhancing anomaly detection and decision making.

### 2.5. LSSVM

To classify higher-dimensional data with a limited number of samples, this study used an LSSVM as a top-level classifier model for DBN [42]. The LSSVM method uses a least squares approach to transform the inequality constraints into equality constraints present in a traditional support vector machine. This transformation simplifies the training by transforming it into linear equations, reducing the complexity of the algorithm. Equation (6) expresses the problem-solving equation of classification as

(6) $$\underset{\left(w_{c},r,v\right)}{\min}F\left(w_{c},r,v\right)=\frac{1}{2}w_{c}^{T}w_{c}+\frac{1}{2}w\sum_{a=1}^{m}v_{a}^{2}$$

(7) $$z_{a}\left[w_{c}^{T}\varphi \left(y_{a}\right)+r\right]=1-v_{a}$$

where,

(8) w>0, a=1,2,3…,m

The term $w_{c}$ denotes the weight coefficient vector, v represents the deviation vector, r signifies the threshold, w is the weight or penalty factor, $z_{a}$ is the category label, and *φ* denotes the kernel function that ensures linear separability of samples in a higher-dimensional space. The introduction of the Lagrange function helps resolve the function’s maximum condition, leading to LSSVM classification as shown in Equation (9).

(9) $$z\left(y\right)=sign[\sum_{a=1}^{m}\alpha_{a}z_{a}K\left(y,y_{a}\right)+r]$$

where y represents the track leak feature vector extracted by DBN, and $K\left(y,y_{a}\right)$ is the kernel function, with the radial basis kernel function chosen specifically for the proposed approach. Equation (10) shows the radial kernel function mathematically, with $\sigma^{2}$ denoting the kernel function parameter.

(10) $$K\left(y_{a},y_{b}\right)=e^{-2|(y_{a}-y_{b})|\sigma^{2}}$$

The weight term w regulates the penalty for errors beyond the error sample. A higher value for w enhances adaptability but increases the risk of overfitting. Conversely, a smaller w reduces the complexity of the model, making it more susceptible to underfitting. The kernel function parameter $\sigma^{2}$ influences the dimensionality of the output space.

### 2.6. Genetic Algorithm

**Figure 4:**
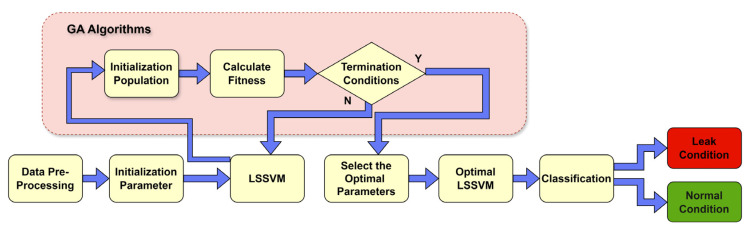
Flowchart for Genetic Algorithm.

The GA function relies on the concept of “survival of the fittest”, based on the major principles of Charles Darwin’s theory of evolution. GA uses the key steps of selection, crossover, and mutation, which are known for their effectiveness in ensuring resilience and achieving global optimization. In the proposed approach, GA is used to refine the penalty factor parameters and kernel function for the LSSVM. The effectiveness of these adjustments can be evaluated using the classification accuracy observed for the test dataset, which serves as the fitness benchmark during the optimization of LSSVM parameters. The optimization process facilitated by the algorithm is depicted in Figure 4 as a flowchart.

In the current study, a GA is employed to optimize the hyperparameters of the SVM used for leak detection. GA begins by initializing a population of candidate solutions, each representing different values for the hyperparameters (C), gamma (γ), and kernel type. Through iterative generations, GA evaluates the fitness of each candidate based on the accuracy of an SVM trained with those parameters. The algorithm uses selection, crossover, and mutation to evolve the population, continually improving the solutions. The best-performing hyperparameters from the final generation are then selected to train the final SVM model, ensuring optimal performance.

## 3. Experimental Setup

**Figure 5:**
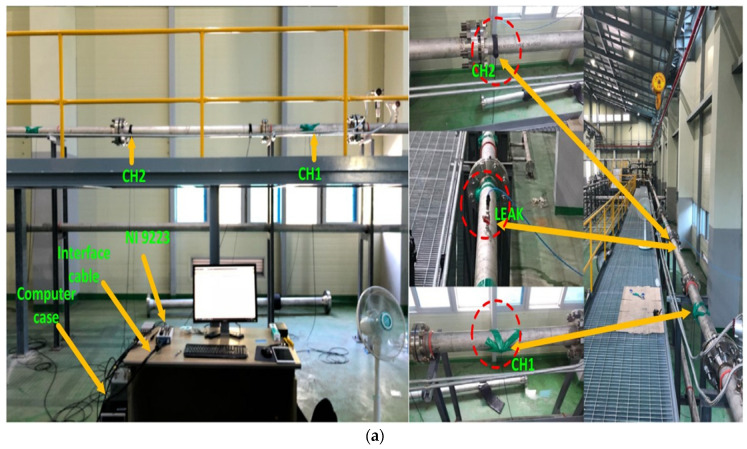

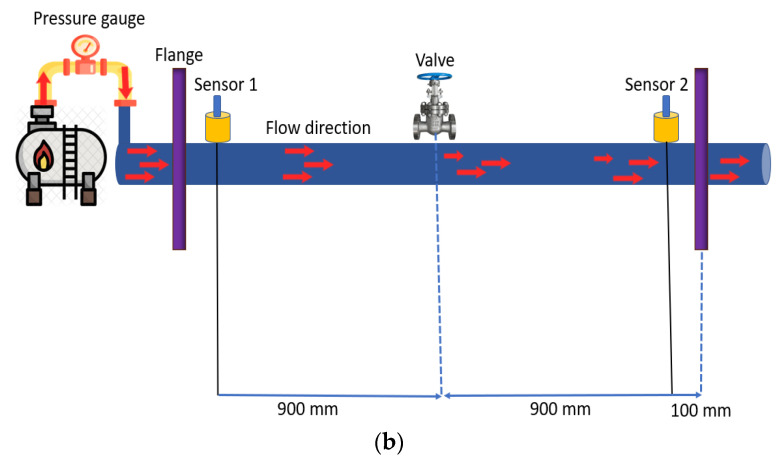
(**a**) Experimental Setup Overview for Acoustic Emission-Based Leak Detection (**b**) Schematic Visualization for AE-Based Leak Detection.

Figure 5a,b depict the methodology and setup of the study. The experiment used a stainless-steel water pipeline with an outer diameter of 114.4 mm and a thickness of 6 mm to simulate pipeline leakages. R15I-AST sensors from Mistras Group, Inc. (New Jersey, NJ, USA) were fixed on the pipeline to detect AE signals, which were then captured at a sampling rate of 1 MHz using a National Instruments NI-9223 data acquisition system connected to a PC in Austin, TX, USA. To create leaks of different sizes, holes were drilled into the pipeline with an electric drill. A fluid control valve was attached at the hole’s location to regulate fluid flow. Water was used for testing due to its non-hazardous nature, facilitating the development of secure and controlled leak scenarios.

**Figure 6:**
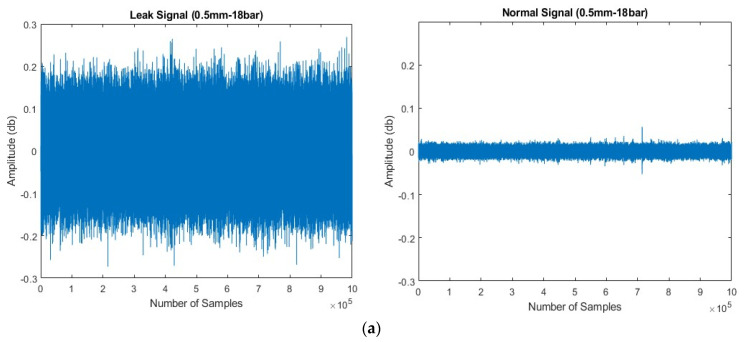

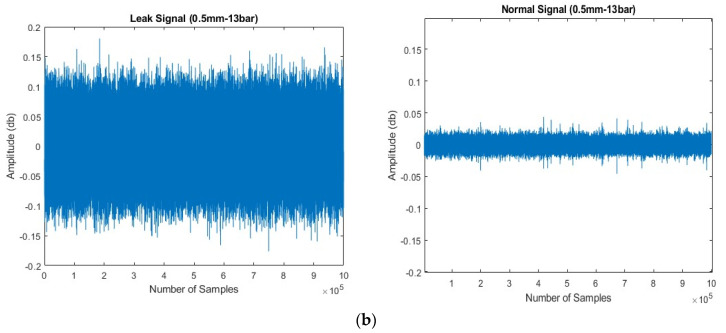
AE Signals Comparison for Leak and Non-leak Conditions (**a**) 18 bar pressure (**b**) at 13 Bar Pressure.

Leak simulations were conducted at two pressure levels: 13 and 18 bar. The testing procedure began with the valve completely closed, involving data collection for 2 min during normal pipeline operation. The valve was then opened to simulate a leak 1 mm in size, extending data collection for an additional 4 min. Valve closure ensured stabilization of the pipeline’s flow. This procedure was repeated for pressure levels of 13 and 18 bar while keeping the leak size consistent. The length of each signal is 1 s, acquired with a sampling frequency of 1 MHz. A total of 360 signal samples were obtained, comprising 120 samples during normal operation and 240 samples during the leak state, from each test. Figure 6 shows AE signals captured during both normal and leaky pipeline conditions. For the safety of the personnel performing the data collection, any fluid escaping from the pipeline was safely directed into a receptacle through a hose.

## 4. Experimental Data Collection

The setup for the experiment consisted of a pipeline designed to transport fluids and gases, with a pressure of either 13 or 18 bar regulated by a centrifugal pump (CP). The procedure began with setting the system pressure to 13 bars and putting the valve in a closed position, which allowed the collection of baseline operational data. Then, with the pressure still at 13 bar, the leak valve was opened gradually to a width of 1 mm, facilitating the compilation of information for dataset A. After completing the data-gathering exercise at 13 bar, the leak valve was shut and the system pressure was increased to 18 bar, reflecting standard operational conditions. Following this, the pressure was maintained at 18 bar, but the position of the valve was adjusted to a narrower opening of 0.3 mm, leading to the creation of dataset C. This method was repeated to generate datasets B and D, respectively. To ensure safety, the valve aperture was restricted to a maximum of 0.5 mm for data collection. Table 2 details the characteristics and parameters of each dataset.

### Proposed Method: Surveillance Zone Identification

According to ISO standard 18211:2016, assessing the surveillance zone requires evaluating the attenuation properties of AE signals produced by noise from the AE source. This assessment is performed prior to collecting data from the AE sensor. In audio engineering, attenuation refers to the reduction in signal intensity, generally measured in decibels (dB). The attenuation characteristics of an AE sensor can be calculated using the following Equation (2) [43].

(11) $$A_{dB}=20\mathrm{Log}\frac{V}{V*}$$

**Figure 7:**
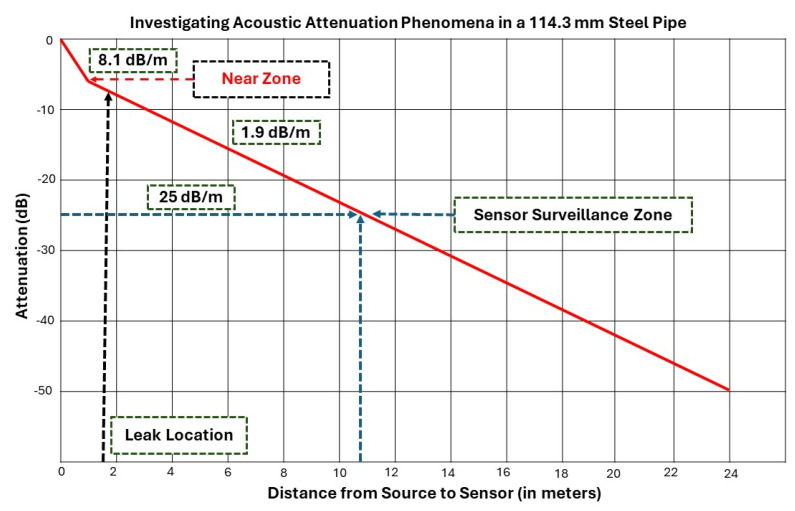
AE Signal Attenuation in 114.3 mm diameter steel pipe.

Equation (2) represents the measured potential (V) and the reference potential (V*). The “measured AE potential” refers to the acoustic emission (AE) signal captured by the AE sensor. In AE terminology, a reference point of 0 dB equates to an AE signal potential of 1 µV at the AE sensor without amplification (Figure 7).

This study utilizes the HSU–Nielsen test as an active AE source to evaluate the attenuation characteristics of the AE sensor. The HSU–Nielsen test involves conducting a pencil lead break test, where a 0.5 mm diameter lead is applied to the pipeline’s surface to generate an acoustic emission event. The AE signals detected in this test are similar to those from natural AE sources, like leaks. Figure 10 shows the attenuation characteristics of a fluid-filled industrial pipeline with an outer diameter of 114.3 mm. The proposed method classified normal and HSU–Nielsen test activities with over 95% accuracy when attenuation in the AE signals collected by the R15I-AST AE sensor was below 25 dB. Figure 9 shows this attenuation level at 10.9 m. Thus, the method can provide surveillance for up to 10.9 m.

## 5. Result Comparison and Discussion

The configuration of training and testing data is important in evaluating the efficiency of the proposed methodology. For training purposes, a dataset comprising 1 mm leaks under fluid pressures of 13 bar and 18 bar was utilized. In the evaluation phase, three distinct datasets were employed, featuring leak sizes of 0.5 mm, 0.7 mm, and 1 mm, all under 7 bars of pressure. The dataset for this research consisted of 960 samples. Among these, half were obtained during normal operating conditions, whereas the remaining half was collected during the leak state. During model training, 80% of the samples were randomly chosen, while the remaining 20% were reserved for testing. Considering the importance of reliability and consistency of the findings, each dataset underwent 20 tests, representing the number of experiments conducted. This approach to dataset selection and testing methodology helps validate the robustness and generalizability of the proposed method.

### Suggested Approach: Comparison of Performance

The method introduces a DBN-GA-LSSVM architecture for analyzing ELIS to detect leaks effectively. This framework distinguishes between broad leak indicators, which capture general patterns and characteristics, and leak-specific indicators, which are designed to pinpoint leak-related information. Combining a DBN with a GA, the approach significantly improves the LSSVM’s ability to conduct sophisticated time–frequency analyses for leak detection. To compare the method’s efficacy with that of traditional techniques, performance indicators such as recall, precision, F-1 score, and accuracy were chosen. These metrics are critical for evaluating a classification algorithm’s performance and the precision of its detections. The calculations for these metrics are detailed in Equations (12)–(15).

(12) $$\mathrm{Recall}\;=\frac{\sum_{\alpha }^{A}\;n_{\alpha }\times \left(\frac{TP_{\alpha }}{TP_{\alpha }+FN_{\alpha }}\right)}{N},$$

(13) $$\mathrm{Precision}\;=\frac{\sum_{\alpha }^{A}\;n_{\alpha }\times \left(\frac{TP_{\alpha }}{TP_{\alpha }+FP_{\alpha }}\right)}{N},$$

(14) $$F1=\frac{1}{N}\sum_{\alpha }^{A}\;n_{\alpha }\times 2\times \sum_{\alpha }^{A}\;\left(\frac{{\;\mathrm{Recall}\;}_{\alpha }\times {\;\mathrm{Precisio}\;}_{\alpha }}{{\;\mathrm{Recall}\;}_{\alpha }+{\;\mathrm{Precisio}\;}_{\alpha }}\right),$$

(15) $$\mathrm{Accuracy}\;=\frac{\sum_{a}^{A}\;TP_{\alpha }}{N},$$

The terms $TP_{\alpha }$, $FP_{\alpha }$, and $FN_{\alpha }$ correspond to the classification outcomes for samples within class A, signifying “true positive”, “false positive”, and “false negative” results, respectively. A “false positive” outcome occurs when a sample is mistakenly identified as part of class A when it does not belong to that class. “True positive” outcomes accurately pinpoint samples that are actual members of class A. “False negative” results occur when samples that should be classified under class A are incorrectly assigned elsewhere. Here, A represents the total count of distinct classes in the dataset and $n_{\alpha }\;$ symbolizes the total samples from class A. The sum of $TP_{\alpha }$ and $FN_{\alpha }$, represented as $n_{\alpha }$, indicates the entire count of samples that truly belong to class A. Meanwhile, the sum of $FP_{\alpha }$ and the subtraction of $n_{\alpha }\;$ from the overall sample count show the number of samples incorrectly classified as part of class A.

In the classification algorithm, N is the total number of data samples in the testing sets. This parameter plays an important role in assessing the algorithm’s performance and evaluating classification accuracy. Accuracy improves as the number of data samples increases, ensuring a more precise evaluation of the algorithm’s effectiveness. It is therefore essential to ensure that the testing sets contain enough samples to provide precise assessments of the performance of the classification algorithm.

Here, we discuss a novel approach to detecting leaks in pipelines using AE data. While deep learning techniques have been effective in feature extraction, the effect of background noise in a signal on model performance remains problematic. To address this concern, we propose a novel model that uses several filters and t-SNE to extract discriminant features from the data. To evaluate the effectiveness of the proposed approach, it was compared with two other state-of-the-art models used in similar applications: the convolutional autoencoder (CAE) and neural network (NN) frameworks, as utilized by Shukla and Piratla (or Masoumeh Rahimi) et al. [44,45] and Ahmad et al. (or Prosvirin) [27]. The former team used a convolutional neural network (CNN) for feature extraction and multilayer classification, while the latter used a combined CNN-CAE to extract discriminative features from complex and noisy AE data. Both models were applied to our dataset, and performance comparisons were conducted.

Shukla and Piratla [45] employed deep learning to identify leaks, using data collected from a leaky plastic tank by a hydrophone. Their approach integrated time, frequency, and time–frequency domain signals into a deep learning CNN model for leak detection. They found that the CNN model was sensitive to the frequency spectrum of the AE signals. Rahimi et al. [44] used comparable techniques on the same dataset, yielding similar results. This methodology was chosen for comparison purposes due to its compatibility with our experimental setup. Upon applying the methodology outlined by each research team to our dataset, the performance metrics shown in Table 3 were achieved. However, we anticipated that these methods might underperform due to strong noise interference in the AE signals. Moreover, the signals exhibited significant non-stationarity, potentially limiting our ability to use the Fourier spectrum to detect leak-related signal changes.

In the second comparison model, Prosvirin et al.’s strategy involved the use of kurtograms, which are derived from preprocessing vibration signals from the CP, alongside a sophisticated NN framework that incorporates both CNN and CAE elements to identify mechanical issues within the CP [46]. This technique was selected for comparison purposes due to the resemblance of its deep neural architecture to that discussed in Ahmad et al. [27], showcasing a similar deep learning application for pipeline leak detection using CWT scalograms. When Ahmad et al.’s methodology was applied to our data, it yielded the accuracy, precision, F-1 scores, and recall for different leak sizes listed in Table 4 The AE signals consisted of persistent background noise in addition to distinct AE hits linked to leaks. AE visual representations such as scalograms and kurtograms highlighted signal fluctuations, but they were partially obscured by background noise. It is therefore important to apply further preprocessing to these visualizations, a step that is emphasized in our proposed method. This additional layer of preprocessing is a key factor responsible for the improved accuracy of our method compared with baselines established by Prosvirin et al. [46] and Ahmad et al. [27]. Finally, the proposed model is also compared with FFT-CNN. An FFT-CNN model for AE leak detection involves converting time-domain AE signals to frequency domain using Fast Fourier Transform (FFT), normalizing, and reshaping the data to fit a CNN, and training the CNN to classify features indicative of leaks. This approach effectively combines FFT for feature extraction with CNN’s pattern recognition capabilities. Using the same dataset for this model, the classification was carried out. However, limitations include potential overfitting due to the complexity of the CNN, sensitivity to noise in the AE signals affecting FFT accuracy, and the need for a large, well-labeled dataset for effective training and generalization.

The comparison was based on four performance parameters, with each model subjected to 20 sets of experiments across three distinct datasets. The findings consistently show that the proposed technique outperformed both the CNN and the CAE-NN models in all experiments and across all performance metrics. The proposed model demonstrated superior performance by extracting more discriminative features using ELIS, making it possible to propose a comprehensive model of a DBN-GA-LSSVM framework and t-SNE and achieve a greater degree of separation between different data classes. Overall, this study makes a major contribution to the field of leak detection in pipelines by introducing a novel approach that addresses the challenge posed by signal noise in deep learning models. The results suggest that the proposed approach can improve the reliability and accuracy of leak detection systems, with critical implications for pipeline safety and environmental protection. Below are the performance parameter results for the three models.

In this study, the proposed approach and a comparison method were rigorously evaluated using a comprehensive set of metrics, and their results were compared. The suggested technique consistently outperformed the comparison methods across all datasets, exhibiting greater accuracy, precision, F-1 scores, and recall of 99.66%, 99.59%, 99.59%, and 99.59%, respectively. Moreover, the suggested techniques, the Rahimi and Ahmad models, were also benchmarked against a reference strategy, surpassing it across all datasets with notable accuracy, precision, F-1 scores, and recall of 96.95%, 97.35%, 97.42%, and 97.20% and 96.72%, 96.98%, 97.02%, and 96.92%, respectively. These findings underscore the superior performance of the suggested method in comparison with both the comparison and reference approaches, establishing its efficacy in leak detection tasks. Table 3 lists the average performance metrics of the proposed model and the other two cited models.

**Figure 8:**
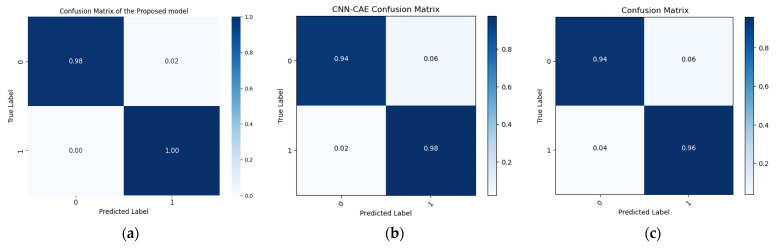
Confusion matrix comparison of the suggested model (**a**) with the Rahimi (**b**) and Ahmad (**c**) models (leak size = 1.0 mm).

**Figure 9:**
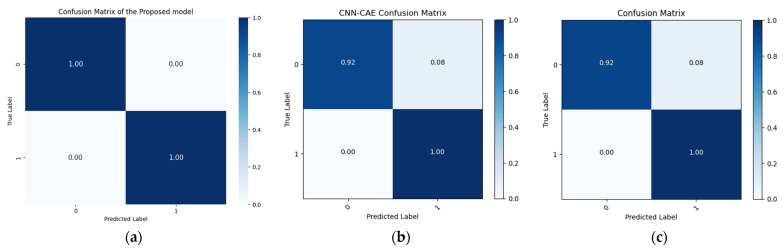
Confusion matrix comparison of the suggested model (**a**) with the Rahimi (**b**) and Ahmad (**c**) models (leak size = 0.7 mm).

**Figure 10:**
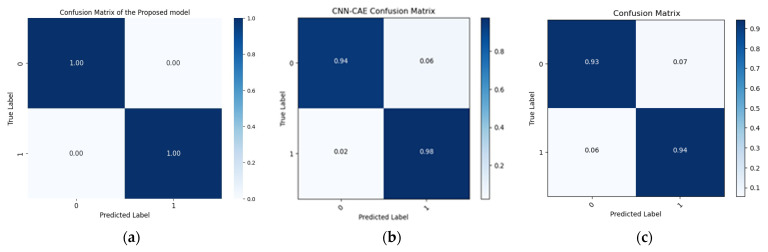
Confusion matrix comparison of the suggested model (**a**) with the Rahimi (**b**) and Ahmad models (**c**) (leak size = 0.5 mm).

**Figure 11:**
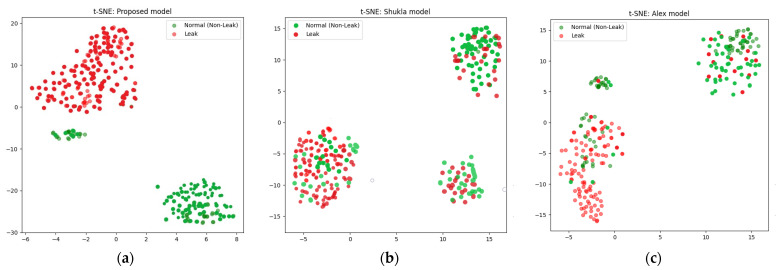
t-SNE comparison of the suggested model (**a**) with the Rahimi (**b**) and Ahmad (**c**) models (leak size = 1 mm).

**Figure 12:**
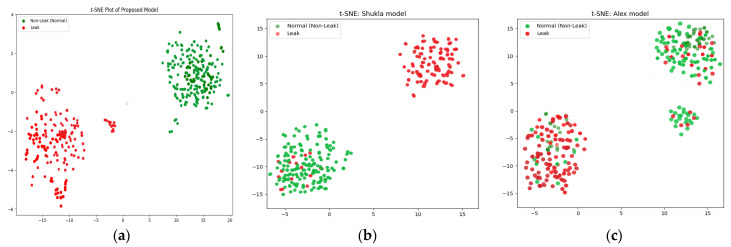
t-SNE comparison of the suggested model (**a**) with the Rahimi (**b**) and Ahmad (**c**) models (leak size = 0.7 mm).

**Figure 13:**
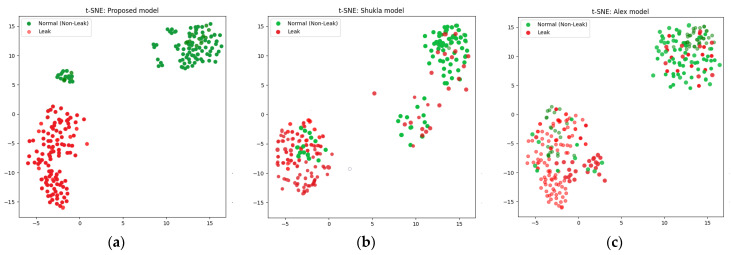
t-SNE comparison of the suggested model (**a**) with the Rahimi (**b**) and Ahmad (**c**) models (leak size = 0.5 mm).

The results produced by the proposed method are more accurate than those associated with the referenced approaches, mainly because they were more accurate and consistent for the leak state and normal operating conditions of the pipeline, as illustrated by the confusion matrices in Figure 8, Figure 9 and Figure 10, as well as the comparisons of the t-SNE visualizations in Figure 11, Figure 12 and Figure 13. Unlike recently cited methods, which struggled to accurately assess pipeline conditions due to such characteristics being either too scattered or not sufficiently distinctive between normal and leak conditions, the proposed method excelled in discriminating between these states. Furthermore, an ablation study was carried out to determine the effectiveness of the proposed model. In this study, CWT scalograms were used for training purposes of the DBN, and then we trained the SVM with default hyperparameters instead of those optimized by GA (CWT-DBN-SVM). The performance was notably lower, underscoring the critical role of GA in fine-tuning the model for optimal performance.

The proposed method involved analyzing the pipeline’s signals in MATLAB R2022b software. The procedure for extracting features from the AE images and then classifying them required approximately 920 s across five trials, conducted on a computer equipped with a 4.2 GHz processor and 24 GB of RAM.

## 6. Conclusions

Implementation of an advanced deep learning model that incorporates a CWT for preprocessing images with NLM and AHE resulted in novel scalograms known as ELIS. These scalograms were further used as input for an optimized DBN combined with a GA, and LSSVM demonstrated exceptional effectiveness in detecting leaks within pipeline systems. This approach underscores the model’s ability to precisely differentiate between leak and non-leak conditions, achieving an unprecedented accuracy of up to 99.60%. In addition to its outstanding accuracy, the model outperformed existing methodologies across various performance metrics, establishing a new standard in pipeline monitoring and leak detection. The integration of ELIS image analysis with a sophisticated DBN-GA-LSSVM framework not only represents a significant advancement in the integrity and safety of fluid transportation systems but also showcases the potential for real-time industrial applications. This highlights the transformative impact of combining advanced signal processing with deep learning and optimization algorithms, presenting a promising avenue for the development of pipeline leak detection.

Future research should focus on enhancing leak localization in pipelines by integrating advanced sensing and signal processing technologies. Furthermore, the proposed method will be validated for more severe leaks in the pipelines using different leak sizes and pressure conditions. Refining the current model and exploring new technologies can improve monitoring and reduce infrastructure risks. This advancement will lead to more efficient and safer pipeline systems.

## Figures and Tables

**Table 1:** Architecture table outlining the layers and configurations of the Deep Belief Network (DBN).

Layer	Output Shape	Activation	Regularization	Dropout
Dense	(512,)	ReLU	L2 (0.01)	None
Batch Normalization	(512,)	-	-	None
Dropout	(512,)	-	-	0.5
Dense	(512,)	ReLU	L2 (0.01)	None
Batch Normalization	(512,)	-	-	None
Dropout	(512,)	-	-	0.5
Dense	(256,)	ReLU	L2 (0.01)	None
Batch Normalization	(256,)	-	-	None
Dropout	(256,)	-	-	0.5
Dense	(128,)	ReLU	L2 (0.01)	None
Batch Normalization	(128,)	-	-	None
Dropout	(128,)	-	-	0.5
Dense	(64,)	ReLU	L2 (0.01)	None
Batch Normalization	(64,)	-	-	None
Dropout	(64,)	-	-	0.5
Dense	(1,)	Sigmoid	None	None

**Table 2:** Detailed Description of Data Acquisition Configuration and Setup.

Dataset	Fluid Pressure (Bars)	Size of Leak (mm)	Duration (min)	Number of Samples Condition (Normal/L = Leak)
A	Water: 13	1.00	6	120/240
B	Gas: 13	0.50	6	120/240
C	Water: 18	0.70	6	120/240
D	Gas:18	0.50	6	120/240

**Table 3:** Performance evaluation: Comparing average metrics of the proposed model versus Rahimi et al. [44,45] and Ahmad et al. [27,46].

Models	Accuracy	Precision	F-1 Score	Recall
Proposed	99.66	99.59	99.59	99.59
Shukla et al.	96.95	97.35	97.42	97.20
Ahmad et al.	96.72	96.98	97.02	96.92
FFT-CNN	95.13	95.53	95.09	95.04

**Table 4:** Performance evaluation: Comparing performance metrics of the proposed model with those of Rahimi et al. [44,45] and Ahmad et al. [27,46].

Models	Accuracy			Precision			F-1 Score			Recall		
	1 mm	0.7 mm	0.5 mm	1 mm	0.7 mm	0.5 mm	1 mm	0.7 mm	0.5 mm	1 mm	0.7 mm	0.5 mm
Proposed	99.43	100.00	99.56	99.37	100.00	99.41	99.43	100.00	99.36	99.43	100.00	99.36
Rahimi et al.	95.43	97.63	97.81	96.74	97.68	97.65	96.94	97.73	97.61	96.74	96.93	97.95
Ahmad et al.	96.40	97.22	96.56	96.86	97.12	96.96	96.82	97.48	96.78	96.40	97.49	96.88
FFT-CNN	96.67	95.38	93.33	96.69	95.89	94.02	96.67	95.26	93.33	96.64	95.22	93.27

## Data Availability

The data were obtained from the industry. Owing to the privacy policy of the industry, the data are not publicly available.

## Nomenclature

DL	deep learning
AE	acoustic emission
ANN	artificial neural network
NLM	non-local mean
AHE	adaptive histogram equalization
GA	genetic algorithm
LSSVM	least squares support vector machine
CWT	continuous wavelet transform
CNN	convolutional neural network
